# Factors Associated with an Outbreak of COVID-19 in Oilfield Workers, Kazakhstan, 2020

**DOI:** 10.3390/ijerph19063291

**Published:** 2022-03-10

**Authors:** Dilyara Nabirova, Ryszhan Taubayeva, Ainur Maratova, Alden Henderson, Sayagul Nassyrova, Marhzan Kalkanbayeva, Sevak Alaverdyan, Manar Smagul, Scott Levy, Aizhan Yesmagambetova, Daniel Singer

**Affiliations:** 1 United States Centers for Disease Control and Prevention, Central Asia, Almaty A25X0T1, Kazakhstan; dps4@cdc.gov; 2 Central Asia Field Epidemiology Training Program, Almaty A25X0T1, Kazakhstan; rtaubayeva19@gmail.com (R.T.); ainura.maratova@gmail.com (A.M.); saya_82@bk.ru (S.N.); kalkanbaevamarzhan@gmail.com (M.K.); 3 School of Public Health, Asfendiyarov Kazakh National Medical University, Almaty A05H2A6, Kazakhstan; 4 Department of Sanitary and Epidemiological Control of Atyrau Region, Division of Sanitary and Hygienic Control and Supervision of Municipal Facilities, Atyrau E04G2K2, Kazakhstan; 5 Department of Sanitary and Epidemiological Control of Eastern Kazakhstan Region, Division of Hospital-Acquired Infections Control, Ust-Kaman 070000, Kazakhstan; 6 United States Centers for Disease Control and Prevention, Workforce and Institute Development Branch, Atlanta, GA 30329, USA; akh0@cdc.gov; 7 Department of Sanitary and Epidemiological Control of Western Kazakhstan Region, Division of Epidemiological Control of Infectious and Parasitic Diseases, Uralsk L02A8F6, Kazakhstan; 8 Department of Sanitary and Epidemiological Control of Eastern Kazakhstan Region, Division of Epidemiological Control of Infectious Diseases, Altay F42D3P7, Kazakhstan; 9 Manoogian Simone College of Business and Economics, American University of Armenia, Yerevan 0019, Armenia; s.alaverdyan@iset.ge; 10 Scientific and Practical Center of Sanitary-Epidemiological Examination and Monitoring, Branch of the National Center for Public Health, Almaty A15G7D2, Kazakhstan; manarka@mail.ru; 11 Chevron Products UK Limited, London E14 4HA, UK; scottlevy@chevron.com; 12 Ministry of Health, Committee of Sanitary and Epidemiological Control, Nur-Sultan 010000, Kazakhstan; yesmagambetovaa@dsm.gov.kz

**Keywords:** COVID-19, SARS-CoV-2, oilfield, pandemic, occupational setting, individual factors, environmental factors, worker safety, FETP, Kazakhstan

## Abstract

From March to May 2020, 1306 oilfield workers in Kazakhstan tested positive for SARS-CoV-2. We conducted a case-control study to assess factors associated with SARS-CoV-2 transmission. The cases were PCR-positive for SARS-CoV-2 during June–September 2020. Controls lived at the same camp and were randomly selected from the workers who were PCR-negative for SARS-CoV-2. Data was collected telephonically by interviewing the oil workers. The study had 296 cases and 536 controls with 627 (75%) men, and 527 (63%) were below 40 years of age. Individual factors were the main drivers of transmission, with little contribution by environmental factors. Of the twenty individual factors, rare hand sanitizer use, travel before shift work, and social interactions outside of work increased SARS-CoV-2 transmission. Of the twenty-two environmental factors, only working in air-conditioned spaces was associated with SARS-CoV-2 transmission. Communication messages may enhance workers’ individual responsibility and responsibility for the safety of others to reduce SARS-CoV-2 transmission.

## 1. Introduction

The novel coronavirus disease 2019 (COVID-19) pandemic has disrupted lives and economies worldwide. Between the initial announcement of the COVID-19 outbreak in Wuhan, China and July of 2021, there were over 180 million cases and 3.9 million deaths reported globally [1]. The virus spreads by respiratory droplets and is found in many occupational settings [2,3]. To prevent transmission, many countries changed business practices by reducing in-person workforces and increasing remote work [4,5].

Large COVID-19 outbreaks continued to be reported in health and social care settings, leisure and transport services, meat and poultry plants, and factories [6,7,8,9]. A lack of physical distancing and ventilation are the key drivers of disease transmission, with shift work posing additional environmental risks [10,11]. Super-spreading transmission occurs when a combination of host, environmental, and viral risk factors are present [10,11]. When outbreaks occur in occupational settings, determining how much SARS-CoV-2 transmission occurs in the occupational setting and how much is associated with household, social, or transport exposures is challenging [12].

Between March and May of 2020, the Atyrau region of Kazakhstan reported 1306 COVID-19 patients. This was the third largest hotspot for COVID-19 in the country with an incidence rate of 203 cases per 100,000 population during this period. Of these patients, 1074 (82%) worked at the Tengizchevroil oil facility in Tengiz, and 90% of these cases were asymptomatic [13].

Tengiz is the sixth largest oilfield in the world with an estimated 25 billion barrels of oil. In May 2020, due to increasing COVID-19 cases, the Kazakhstan Ministry of Health (MoH) considered suspending production at the oilfield. In response, the oil facility demobilized 20,000 workers, or two-thirds of the workforce. Approximately 13,000 workers continued to work at the oilfield [14]. The number of COVID-19 cases at the oilfield site increased to 2661 by 29 July 2020. A team comprising the U.S. Centers for Disease Control and Prevention (CDC) staff and Kazakhstan MoH employees associated with the Field Epidemiology Training Program investigated the individual and environmental factors contributing to COVID-19 among these oilfield workers.

## 2. Materials and Methods

### 2.1. Study Design

We conducted a concurrent case-control study among Tengizchevroil (TCO) oilfield workers who worked on-site between 1 June and 15 September 2020.

### 2.2. Specific Setting

TCO is one of the largest producers and marketers of oil and gas in Kazakhstan. It is a limited liability partnership (LLP) between four international companies and one national company to develop the Tengiz and Korolevskoye oilfields in the Atyrau region of Kazakhstan [15].

Oilfield workers are housed in 94 shift camps or villages for four or eight consecutive week-long rotations. TCO employees are quartered in two camps, Shanyrak and TCO Village, with contractors, subcontractors, and employees of other private companies in the remaining 92 camps.

COVID-19 outbreaks were registered in 34% (32/94) of the shift camps. For this study, eight shift camps with the highest COVID-19 incidence in June and July 2020 were selected: TCO Village, Shanyrak, Bolashak, New Tengiz, Senimdi Kurylis, Denholm Zholdas, Karat, and Birlik.

After reducing the on-site workforce, TCO introduced additional COVID-19 mitigation measures for its employees and contractors. All workers underwent a mandatory five or ten day pre-rotation quarantine upon arrival in their rotation at the shift camp. The workforce received real-time reverse transcription polymerase chain reaction (rRT-PCR) tests for the qualitative detection of SARS-CoV-2 at the start and end of their pre-rotation quarantine. All workers with a positive SARS-CoV-2 rRT-PCT test, regardless of symptoms, were hospitalized in TCO or in Atyrau City.

After their pre-rotation quarantine, workers were housed at the dormitories in single- or multiple-occupancy rooms. Most residents of multiple-occupancy rooms shared bathrooms, while single rooms included en-suite bathrooms. Meals were served in shift camp canteens that could accommodate 200 to 960 people. Workers with mild COVID-19 disease identified during rotation work were isolated in the TCO hospitals. Workers with moderate and severe COVID-19 disease were hospitalized to Atyrau City. Contacts of these patients were quarantined in single-occupancy rooms in the shift camps with arranged “door-to-door” meal delivery.

Other mitigation measures included thermal screening of employees entering buildings, mandatory facemask usage, 1.5 m physical distancing requirements, and hand sanitizers placed widely at dormitories, canteens, and workstations, as well as daily PCR testing for SARS-CoV-2 of 5% of a random selection of employees at the shift camps. Multiple-occupancy rooms were not fully occupied to comply with physical distancing requirements. Video cameras in workstations, dormitories, and canteens were used to monitor compliance with the mitigation measures. Additionally, bans on gatherings, the disinfection of vehicles and premises, and changes to catering schedules were implemented.

### 2.3. Study Participants

Employees of the selected eight shift camps that identified as positive for SARS-CoV-2, regardless of COVID-19 symptoms, during their shift work between June and September 2020 were contacted and included as case patients. The case patients also included all workers hospitalized for COVID-19 treatment during the study period in TCO and Atyrau City. Two controls per one case patient were randomly selected among SARS-CoV-2 negative employees that were working or living in the same shift camps during the same rotation period as the case patients. All controls were tested before starting their shift at TCO and had not been diagnosed with COVID-19 infection before enrolling into this study.

### 2.4. Sources and Data Variables

TCO distributed information about the purpose of the study to their employees and informed consent was obtained prior to their participation in this study. Five FETP residents piloted a standardized, structured CDC questionnaire consisting of 123 questions and interviewed the study participants following the pilot. We measured the questionnaire’s internal reliability or consistency using Cronbach’s Alpha test [16]. The Cronbach’s Alpha reliability for 123 items was + 0.786, indicating an appropriate questionnaire internal consistency.

Participants were interviewed telephonically to establish individual and environmental factors that might contribute to virus transmission. The individual factors were socio-demographic characteristics, knowledge, beliefs, and personal practices that included practices outside of working hours, which could not be controlled by the employer. Environmental factors were the working and living conditions that could be mandated by the employer. The average time of interview was about 30 min per person.

### 2.5. Sample Size

Sample size calculations were performed on the “Open Source Epidemiologic Statistics for Public Health” platform (www.OpenEpi.com, updated 6 April 2013). The intent was to recruit 296 cases and 590 controls, assuming an 8% prevalence of multiple risk factors at individual and setting levels for COVID-19 among the controls (unpublished data, Kazakhstan), a two-sided confidence level set at 95%, a 5% margin of error, an odds ratio (OR) of 2 with 80% power, and a 20% adjustment for non-response and confounding [17].

### 2.6. Statistical Methods

Data were analyzed using Stata version 16 (StataCorp LP, College Station, TX, USA) and Epi Info version 7.2.4.0 (CDC, Atlanta, GA, USA). Individual and environmental characteristics were summarized as proportions. The Chi-square test was used to determine if the difference between proportions was statistically significant.

In addition to the descriptive analysis, bivariate logistic regression analysis was used to calculate the OR and the 95% confidence interval (CI) for each risk factor. A *p*-value of < 0.05 was considered statistically significant. Variables which had biologically plausible association with COVID-19 and were relevant to the transmission of SARS-CoV-2 infection were entered into a multivariable logistic regression model. Multivariable logistic regression analysis was completed to generate adjusted ORs (aOR) and 95% CIs. Multicollinearity was assessed by the Generalized Variance Inflation Factor (GVIF) [18,19].

Stratified Mantel–Haenszel analysis was performed to assess for confounding and the difference of the associations of the covariates across the shift camp strata. A Breslow–Day *p*-value and two log likelihoods were obtained to test for significant effect modification. A likelihood ratio test was later calculated based on the likelihoods of two regression equations: (1) an equation containing all the variables; and (2) an equation containing all the variables plus an interaction variable of the variable in the corresponding row and a variable showing the shift camp strata. For both tests, if the *p*-value was < 0.05, we rejected the null hypothesis and concluded that the ORs for the variable were different across regions.

## 3. Results

### 3.1. Recruitment/Response of Participants

**Figure 1:**
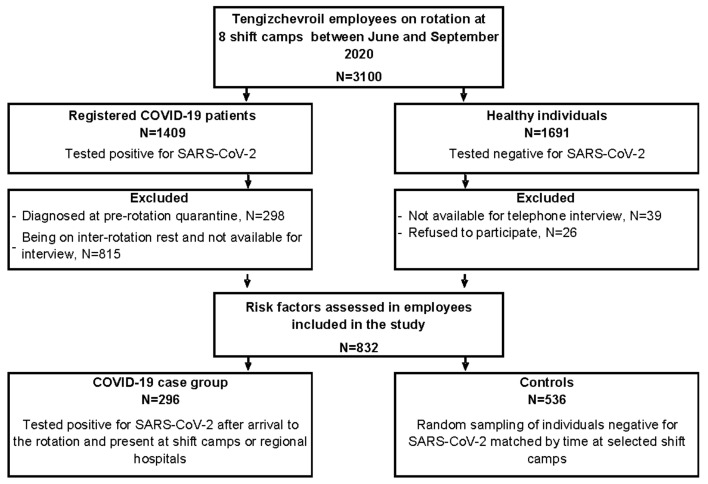
Flow chart of the participants included in the case-control study of factors associated with severe acute respiratory syndrome coronavirus 2 infection in Tengizchevroil, Kazakhstan during June–September 2020.

Between June and September 2020, 1409 (45%) TCO oil workers tested positive for SARS-CoV-2 at the studied camps. We excluded from the study 815 (58%) COVID-19 patients who were off rotation, and thus not available for the interview, and 298 (21%) workers who were identified as positive for COVID-19 prior to arriving to their work rotation (Figure 1). A total of 296 eligible COVID-19 patients were identified during their rotation in the selected camps and all of them agreed to participate in the study as case patients (Table 1). Of the 596 potential controls, we enrolled 536 (90%) participants, as 39 (7%) of the potential controls were unavailable for interview and 26 (4%) refused to participate.

### 3.2. Baseline Characteristics of Case Patients and Controls

Among case patients and controls, there were more men (627, 75%) in the study and 527 (63%) of the participants were less than 40 years of age. There were no statistically significant socio-demographic characteristic differences between case patients and controls, except for education (*p* = 0.003) (Table 1). More than half of the study participants, or 532 (64%), had high school or vocational education, but 125 (42%) of the cases had university degrees compared to 175 (33%) controls (*p* = 0.003). Among the case patients, 168 (56%) were asymptomatic, 95 (32%) exhibited mild disease, and 36 (12%) had moderate to severe COVID-19.

### 3.3. Bivariate Analysis

Our bivariate analysis of the twenty studied individual factors showed a significant positive association between having a positive SARS-CoV-2 test and higher education, distant travel before coming to the rotation, social interaction with peers outside of working hours, belief that COVID-19 is not a serious issue, rare or non-use of hand sanitizer, or the use of disposable surgical masks. Of those who used surgical masks, 17% (139) were using one or two masks daily during a 12 hour work shift (Table 2). Those who received information about COVID-19 symptoms, believed that asymptomatic COVID-19 was contagious, believed they should use masks outdoors or in public places, or used fabric facemasks had lower odds of contracting COVID-19.

Among the twenty-two studied environmental factors, a significant positive association was observed between having a positive SARS-CoV-2 test and a pre-rotation quarantine of less than eight days, contact with a COVID-19 patient, sharing toilets in the dormitory, and working in an infirmary/clinic, in an office setting, in air-conditioned facilities, or on transport (Table 3). Protection against having a positive SARS-CoV-2 test was observed among those who received training on COVID-19 prevention measures, used gloves in the dormitory, worked outdoors or in the kitchen, had ventilated workstations, maintained a 1.5 m physical distance at work, and lived in dormitories with neighbors. Living with people in the same room was protective and was an unusual finding, but in TCO, people living alone were mainly the quarantined contacts of COVID-19 cases. This variable was not included in the multivariate analysis.

### 3.4. Multivariable Analysis

**Figure 2:**
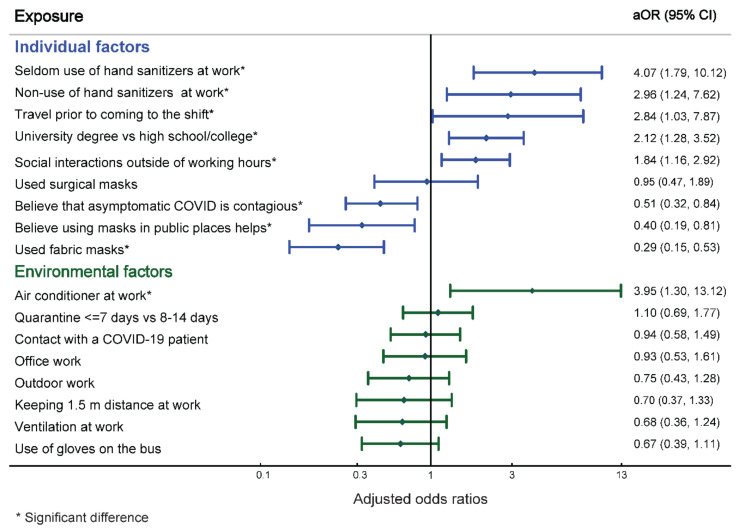
Adjusted odds ratios (aOR) and 95% confidence intervals (CI) for factors associated with the development of the COVID-19 disease in the employees of Tengizchevroil, Kazakhstan during June–September 2020.

Among the individual factors studied, rare use of hand sanitizers (aOR = 4.1, 95% CI = 1.8–10.1), non-use of hand sanitizers at the workplace (aOR = 3.0, 95% CI = 1.2–7.6), travel before arriving to the shift (aOR = 2.8, 95% CI = 1.0–7.9), higher education (aOR = 2.1, 96% CI = 1.3–3.5), and social interaction outside of working hours (aOR = 1.8, 95% CI = 1.2–2.9) were associated with increased odds of COVID-19 acquisition in the multivariable analysis (Figure 2). Belief that asymptomatic COVID-19 infection is contagious (aOR = 0.5, 96% CI = 0.3–0.8), belief they should use facemasks in public places (aOR = 0.4, 96% CI = 0.2–0.8), and the use of fabric facemasks (aOR = 0.3, 96% CI = 0.2–0.5) had a protective association against development of SARS-CoV-2 infection.

Of the environmental factors studied, only air-conditioned premises (aOR = 4.0, 95% CI = 1.3–13.1) was associated with SARS-CoV-2 transmission. No association was observed between pre-rotation quarantine of less than 8 days (aOR = 1.1, 95% CI = 0.7–1.8), exposure to COVID-19 cases (aOR = 0.9, 95% CI = 0.6–1.5), office work (OR = 0.9, 95% CI = 0.5–1.6), work outdoors (aOR = 0.8, 95% CI = 0.4–1.3), or ventilated workstations (aOR = 0.7, 95% CI = 0.4–1.2) and the development of COVID-19. Since all GVIF were less than 5, there was no multicollinearity in the multivariate regression model.

### 3.5. Stratified Analysis

In a stratified analysis, there were significant differences within shift camp strata on the development of COVID-19 among those who used surgical facemasks, respirators, fabric facemasks, gloves at work, knew the COVID-19 symptoms, believed they should use masks in public places and in dormitories, had a pre-rotation quarantine, maintained a 1.5 m physical distance at work, lived in the dormitories with neighbors, were exposed to a COVID-19 case, worked in an office, worked in a kitchen, worked outdoors, worked in air-conditioned premises, and had social interactions outside of working hours (Appendix A, Table A1 and Table A2).

## 4. Discussion

Our study provides evidence that individual factors were the main determinants of SARS-CoV-2 transmission in the oil shift camps in Kazakhstan, with little contribution by environmental factors. Individual factors fell into risk and protective factors. The risk factors were travel before arriving at TCO, infrequent use of hand sanitizers at the workplace, higher education, and social interactions in the shift camps outside of working hours. The protective factors were the beliefs that asymptomatic COVID-19 infection is contagious and that face masks should be used in public places, along with the use of fabric facemasks. The only environmental factor contributing to the transmission of COVID-19 infection in our multivariate model was working in air-conditioned spaces. This is consistent with published research indicating that air-conditioned restaurants in China led to three COVID-19 clusters [20].

We did not find a significant association between environmental factors, such as exposure to COVID-19 cases, working in offices, and working outdoors and in ventilated workstations, and having a positive SARS-CoV-2 test.

Our results are consistent with the global literature on factors associated with SARS-CoV-2 transmission. Studies show a significant correlation between increased number of international COVID-19 cases and passenger volume around the world, prompting countries to close borders and mandate restrictions on international and domestic travel [21,22,23]. To mitigate the effect of travel and prevent further community transmission of COVID-19 infection, many countries, including Kazakhstan, mandated a 14 day post-travel quarantine for arriving passengers in 2020 [24]. 

Likewise, our data indicated that travel was one of the drivers behind the continued spread of COVID-19 to TCO. To mitigate its effect and to diagnose infectious people in a timely manner, strict adherence to at least seven days of pre-rotation quarantine have proven to be effective in published studies [25,26]. 

Unlike travel, the non-significant association of pre-rotation quarantine in our study is not consistent with the literature. We suspect the duration of quarantine explains the difference. At the time, the Government of Kazakhstan recommended a 14 day quarantine after traveling. TCO implemented several different quarantine durations because of their operational needs. The self-reported pre-rotation quarantine duration in our study population ranged between 3 and 14 days. Oil workers were tested the day they started and the day before they left quarantine. 

The inconsistent and short pre-rotation quarantine might underestimate the number of oil workers with rRT-PCR tests that were positive for SARS-CoV-2. When a worker is tested early in quarantine, their test may be falsely negative because of a low viral load [27,28]. This is even more important because TCO had a significantly high number of asymptomatic cases diagnosed later during shift work.

In addition, and consistent with other studies, our findings show that most asymptomatic cases were observed in adults under 40 years of age [29,30]. Younger people are more socially active and those with asymptomatic COVID-19 have increased risk of infection transmission.

Previous research reported that hand sanitizers can prevent transmission SARS-CoV-2 [31,32]. Compliance with hand hygiene recommendations depends on the availability of handwashing stations and hand sanitizers in areas where handwashing is not possible [7]. Noncompliance may be attributed to lower COVID-19 risk perceptions and adverse skin reactions, particularly in males and people with lower income [28].

In our study, the availability of hand sanitizers at TCO was self-reported by 91% of cases and 76% of controls, but compliance was low. Rare or non-use of hand sanitizers among oil workers likely increased the risk of acquiring SARS-CoV-2 infection and is consistent with other studies [33].

In our study, oil workers with higher education were less likely to use hand sanitizer at work compared to oil workers with a high school or vocational degree. The reason for this is unclear, but it is possible that oil workers with higher education did not take mitigation measures seriously, or that monitoring to enforce mitigation measures was not applied uniformly, with more educated workers being less subject to enforcement of recommended practices.

Published data on the effect of education on SARS-CoV-2 transmission are controversial. High educational attainment has been shown to reduce risk of COVID-19 in studies in Mexico and the United Kingdom [34,35], while no association was observed in a study in China [36]. A study in the US showed people with higher education and literacy scores may have polarized views on political and religious matters, and thus may have a negative and careless attitude toward COVID-19 mitigation measures [37]. Like the US, this study identified an increased risk of COVID-19 development among people with higher educational attainment. The association of higher educational attainment with COVID-19 development might also be attributed to the working conditions of people with higher education, which may comprise poorly ventilated or air-conditioned premises and entail more frequent work-related contact with people.

Multiple studies have shown that perceived sociability and a culture of close social, personal, and intimate interaction significantly increases the spread of disease and mortality [38,39]. If the occupational setting limits socialization with each other, people may ignore these rules outside of work, as reported in a study among office workers in the US [40].

Similarly, we found that social contacts in the shift camps outside of work hours contributed to disease spread. People still interact outside of working hours, and they need targeted communication messages that do not prohibit social interactions, but instead suggest ways to safely telecommunicate or interact with peers in-person [41]. Indoor interactions with each other should be made safer by improved air disinfection engineering strategies like mechanical ventilation or upper room germicidal ultraviolet air disinfection (GUV). It has been shown that SARS-CoV-2 is susceptible to GUV, an established technology that has been proven to be a safe, quiet, and cost-effective way to produce the equivalent of 10 to 20 air changes/hour under real-life conditions [42].

Oksanen et al. showed that people with higher institutional trust are more likely to adhere to recommendations and risk mitigation efforts [39]. This is consistent with our findings showing that belief regarding the effectiveness of facemasks or asymptomatic SARS-CoV-2 transmission correlate to protective practices such as the use of fabric masks, and these practices protect against COVID-19. Without institutional trust, deeply rooted beliefs may drive resistance and complicate efforts to control the coronavirus outbreak. To address this, persuasive communication at an appropriate literacy level is needed to appeal to social norms and culture and to develop trust in affected communities.

We observed a protective but non-significant association between surgical mask usage and the development of COVID-19. This finding might be attributed to the fact that one-fifth of the study participants used one or two masks per day, and many used three masks per day, despite a daily 12 hour work shift. This is consistent with studies showing a decreased efficacy of surgical masks after a four hour wearing time and Kazakhstan’s national sanitary–epidemiological guidance in recommending changing surgical masks every three hours [43]. The other possible explanation of the non-significant association of surgical mask usage might be the quality of mask used by the study’s participants [44].

Our final multivariable analysis did not find a significant protective association of maintaining a 1.5 m physical distance on SARS-CoV-2 transmission in the oilfield camps. Our results correlate with recent publications which reported that SARS-CoV-2 may be transmitted by air over distances of at least 8 m from infected people through breathing, speaking, or coughing. The safe distance between people depends on factors such as indoor or outdoor work, ventilation type, air exchange rates, prevalence of recirculated unfiltered air, occupancy, and exposure time, and it is not only dependent on a recommended 1.5 m physical distance [45,46].

We observed significant differences in the effect of individual and environmental factors associated with development of the disease (the use of surgical facemasks, respirators, fabric masks, or gloves at work, and knowledge of COVID-19 symptoms, mask use in public places and in the dormitory, pre-rotation quarantine, physical distancing of 1.5 m at work, having roommates, exposure to a COVID-19 case, office work, kitchen work, outdoor work, working in air-conditioned premises, or social interaction outside of working hours) within different camps that would benefit from targeted mitigation measures.

There are several potential limitations in our study. First, we conducted a study in 8 out of 92 Tengizchevroil camps that reported the highest number of COVID-19 cases by June 2020, and we observed significant differences in the effect of environmental factors associated with development of disease within these camps. Our findings might not be representative of all 92 camps. Second, we were dependent on the verbal responses of workers. Most of the workers in this study were contractors who value their jobs, and thus could be subject to observation or acquiescence and health-seeking biases. Many of them went through infection control and COVID-19 prevention training and might have been hesitant to report negatively on environmental factors or non-compliance in their shift camps. Similarly, workers might have been reluctant to report COVID-19 symptoms to interviewers. However, the shift camps implemented 100% PCR testing for SARS-CoV-2 for all workers during the pre-shift quarantine and daily PCR testing for SARS-CoV-2 for 5% of a random selection of employees at the shift camps. Therefore, we assume that there was no underestimation of cases among our study population.

Finally, our study is a case-control investigation and is subject to some recall and information biases in the assessment of study associations, particularly if those workers who were not available to be interviewed were different from those who were available and on-shift. To avoid that, we conducted a concurrent study matching case patients and controls by time of disease onset in cases and place of work, and we used incident, not prevalent, cases that occurred during the study period, with a structured questionnaire to interview cases and controls.

## 5. Conclusions

This is the first study assessing individual and environmental factors associated with COVID-19 among oil workers. Our study supports the importance of the multilayered approach of individual and environmental interventions introduced in the “Swiss cheese respiratory virus pandemic defense model” by I. Mackay [47]. Each intervention has its holes or drawbacks, and only the cumulative success of multiple layers of interventions, or slices of cheese, can make a difference.

The efficacy of the outbreak response depends on the speed and scale of environmental or governmental intervention and how individuals receive, perceive, and comply with the provided public health and health risk messages [40,48]. Irrespective of how robustly environmental interventions are implemented by an employer, unless we change the beliefs and risk perceptions of people, individual behaviors will not change, thus potentially preventing control of the outbreak.

## Figures and Tables

**Table 1:** General characteristics of the study population in Tengizchevroil, Kazakhstan during June–September 2020.

	COVID-19 Cases, no. (%) N = 296	Controls, no. (%) N = 536	*p*-Value
Sex			
Male	232 (78.4%)	395 (73.7%)	0.133
Female	64 (21.6%)	141 (26.3%)	
Age, years			
20–29	80 (27.0%)	139 (25.9%)	0.333
30–39	119 (40.2%)	189 (35.3%)	
40–49	64 (21.6%)	133 (24.8%)	
50–59	32 (10.8%)	68 (12.7%)	
60+	1 (0.3%)	7 (1.3%)	
Education			
University	125 (42.2%)	175 (32.6%)	0.003 *
Vocational	162 (54.7%)	323 (60.3%)	
High school	9 (3.0%)	38 (7.1%)	
Body mass index, kg/m^2^			
Normal weight (< 24.9)	115 (38.9%)	228 (42.5%)	0.178
Overweight (25.0–29.9)	115 (38.9%)	205 (38.2%)	
Obese > 30	50 (16.9%)	66 (12.3%)	
Missing	16 (5.4%)	37 (6.9%)	
Place of residence			
West Kazakhstan	239 (80.7%)	406 (75.7%)	0.598
South Kazakhstan	35 (11.8%)	86 (16.0%)	
North Kazakhstan	6 (2.0%)	9 (1.7%)	
East Kazakhstan	4 (1.4%)	7 (1.3%)	
Central Kazakhstan	1 (0.3%)	3 (0.6%)	
Other countries	11 (3.7%)	25 (4.7%)	

* Significant difference, Chi-square *p*-value.

**Table 2:** Bivariate model of the individual risk factors associated with novel coronavirus disease among the employees of Tengizchevroil, Kazakhstan during June–September 2020.

Characteristics	Cases, no, (%), N = 296	Controls, no, (%), N = 536	cOR (95% CI) *	*p*-Value
Sex				
Male	232 (78.4%)	395 (73.7%)	1.3 (0.9–1.8)	0.134
Female	64 (21.6%)	141 (26.3%)	1	
Age group				
Less than 36 years	160 (54.1%)	274 (51.1%)	1	
36 years or more	136 (45.9%)	262 (48.9%)	0.9 (0.7–1.2)	0.417
Education				
University degree	125 (42.2%)	175 (32.6%)	1.5 (1.1–2)	0.006 ^†^
Vocational or high school degree	171 (57.8%)	361 (67.4%)	1	
Body mass index (kg/m^2^)				
Normal weight (≤ 24.9)	115 (38.9%)	228 (42.5%)	1	
Overweight (25.0–29.9)	115 (38.9%)	205 (38.2%)	1.1 (0.8–1.5)	0.515
Obese ≥ 30	50 (16.9%)	66 (12.3%)	1.5 (1–2.3)	0.063
Travel before coming to shift				
Yes	17 (5.7%)	12 (2.2%)	2.6 (1.2–5.8)	0.008 ^†^
No	279 (94.3%)	524 (97.8%)	1	
Social interactions outside of working hours				
Yes	138 (46.6%)	159 (29.7%)	2.1 (1.5–2.8)	< 0.001 ^†^
No	158 (53.4%)	377 (70.3%)	1	
Knowledge of symptoms:				
Fever				
Yes	218 (73.6%)	442 (82.5%)	0.6 (0.4–0.8)	0.003 ^†^
No	78 (26.4%)	94 (17.5%)	1	
Cough				
Yes	149 (50.3%)	309 (57.6%)	0.7 (0.6–1)	0.042 ^†^
No	147 (49.7%)	227 (42.4%)	1	
Loss of smell or taste				
Yes	154 (52.0%)	251 (46.8%)	1.2 (0.9–1.6)	0.151
No	142 (48.0%)	285 (53.2%)	1	
Shortness of breath				
Yes	110 (37.2%)	217 (40.5%)	0.9 (0.6–1.2)	0.347
No	186 (62.8%)	319 (59.5%)	1	
Consider COVID-19 to be a nonserious issue				
Not serious or unsure	68 (23.0%)	96 (17.9%)	1.4 (1–2)	0.040 ^†^
Serious	216 (73.0%)	440 (82.1%)	1	
Believe asymptomatic COVID-19 is contagious				
Yes	160 (54.1%)	372 (69.4%)	0.5 (0.4–0.7)	< 0.001 ^†^
No	136 (45.9%)	164 (30.6%)	1	
Believe they should use masks outdoors				
Yes	211 (71.3%)	447 (83.4%)	0.5 (0.4–0.7)	< 0.001 ^†^
No	85 (28.7%)	89 (16.6%)	1	
Believe they should use masks in public places				
Yes	255 (86.1%)	494 (92.2%)	0.5 (0.3–0.8)	0.006 ^†^
No	41 (13.9%)	42 (7.8%)	1	
Believe they should use masks in the dormitory				
Yes	253 (85.5%)	477 (89.0%)	0.7 (0.5–1.1)	0.138
No	43 (14.5%)	59 (11.0%)	1	
Use of N95 respirators				
Yes	34 (11.5%)	57 (10.6%)	1.1 (0.7–1.7)	0.706
No	262 (88.5%)	479 (89.4%)	1	
Use of fabric facemask				
Yes	42 (14.2%)	128 (23.9%)	0.5 (0.4–0.8)	0.001 ^†^
No	254 (85.8%)	408 (76.1%)	1	
Use of surgical facemask				
Yes	246 (83.1%)	410 (76.5%)	1.5 (1.1–2.2)	0.025 ^†^
No	50 (16.9%)	126 (23.5%)	1	
Number of masks changed per day				
5 or more	159 (53.7%)	304 (56.7%)	1	
3–4	72 (24.3%)	150 (28.0%)	1 (0.7–1.4)	0.911
2	43 (14.5%)	61 (11.4%)	1.3 (0.9–2.1)	0.178
1	16 (5.4%)	19 (3.5%)	1.6 (0.8–3.2)	0.174
Use of sanitizer at the work				
Always	26 (8.8%)	100 (18.7%)	1	
Seldom	159 (53.7%)	209 (39.0%)	2.9 (1.8–4.8)	< 0.001 ^†^
Never	111 (37.5%)	227 (42.4%)	1.9 (1.2–3.1)	0.010 ^†^

* cOR, Crude odds ratio; CI, confidence interval; ^†^ Significant difference, Chi-square *p*-value.

**Table 3:** Bivariate model of the environmental factors associated with the novel coronavirus disease among the employees of Tengizchevroil, Kazakhstan during June–September 2020.

Characteristics	Cases, no, (%), N = 296	Controls, no, (%), N = 536	cOR (95% CI) *	*p*-Value
Pre-shift quarantine, N = 589				
Less than 8 days	95 (32.1%)	161 (30.0%)	1.4 (1–2)	0.049 ^†^
8 or more days	98 (33.1%)	235 (43.8%)	1	
Trained on COVID-19 prevention measures				
Yes	248 (83.8%)	486 (90.7%)	0.5 (0.3–0.8)	0.003 ^†^
No	48 (16.2%)	50 (9.3%)	1	
Contact with a COVID-19 patient at work				
Yes	86 (29.1%)	146 (27.2%)	1.4 (1–2)	0.028 ^†^
No	152 (51.4%)	372 (69.4%)	1	
Exposed to a COVID-19 patient with < 1.5 m				
Yes	56 (65.1%)	83 (56.8%)	2.2 (1.2–4.2)	0.016 ^†^
No	17 (19.8%)	55 (37.7%)	1	
Exposed to COVID-19 patient for > 15 min in the room				
Yes	66 (76.7%)	99 (67.8%)	2.2 (1–5)	0.042 ^†^
No	10 (11.6%)	33 (22.6%)	1	
Living in the dormitory				
1–4 roommates	93 (31.4%)	280 (52.2%)	0.4 (0.3–0.5)	< 0.001 ^†^
Alone	201 (67.9%)	240 (44.8%)	1	
Sharing toilet on the floor of the dormitory				
Yes	127 (42.9%)	194 (36.2%)	1.3 (1–1.8)	0.057
No	169 (57.1%)	342 (63.8%)	1	
Individual toilet in the room				
Yes	171 (57.8%)	350 (65.3%)	0.7 (0.5–1)	0.032 ^†^
No	125 (42.2%)	186 (34.7%)	1	
Working in the infirmary/clinic				
Yes	11 (3.7%)	2 (0.4%)	9.7 (2.5–68.8)	< 0.001 ^†^
No	285 (96.3%)	534 (99.6%)	1	
Transport work				
Yes	36 (12.2%)	43 (8.0%)	1.6 (1–2.5)	0.051 ^†^
No	260 (87.8%)	493 (92.0%)	1	
Working in an office				
Yes	88 (29.7%)	115 (21.5%)	1.5 (1.1–2.1)	0.008 ^†^
No	208 (70.3%)	421 (78.5%)	1	
Working outdoors				
Yes	86 (29.1%)	196 (36.6%)	0.7 (0.5–1)	0.028 ^†^
No	210 (70.9%)	340 (63.4%)	1	
Working in a kitchen				
Yes	29 (9.8%)	96 (17.9%)	0.5 (0.3–0.8)	0.002 ^†^
No	267 (90.2%)	440 (82.1%)	1	
Working in a storeroom				
Yes	3 (1.0%)	10 (1.9%)	0.6 (0.1–1.9)	0.343
No	293 (99.0%)	526 (98.1%)	1	
Other work stations ^‡^				
Yes	56 (18.9%)	82 (15.3%)	1.3 (0.9–1.9)	0.179
No	240 (81.1%)	454 (84.7%)	1	
Maintaining 1.5 m distance at work				
Yes	221 (74.7%)	453 (84.5%)	0.4 (0.3–0.7)	< 0.001 ^†^
No	61 (20.6%)	55 (10.3%)	1	
Air conditioner at work				
Yes	17 (5.7%)	7 (1.3%)	4.5 (1.9–12)	< 0.001 ^†^
No	279 (94.3%)	529 (98.7%)	1	
Ventilation system at work				
Yes	32 (10.8%)	103 (19.2%)	0.5 (0.3–0.8)	0.002 ^†^
No	264 (89.2%)	433 (80.8%)	1	
Availability of hand sanitizers at work				
Yes	268 (90.5%)	405 (75.6%)	3.2 (2.0–5.1)	< 0.001 ^†^
No	24 (8.1%)	115 (21.5%)	1	
Use of gloves in the dormitory corridors				
Yes	64 (21.6%)	169 (31.5%)	0.6 (0.4–0.8)	0.002 ^†^
No	232 (78.4%)	367 (68.5%)	1	
Use of gloves on the bus				
Yes	70 (23.6%)	191 (35.6%)	0.6 (0.4–0.8)	< 0.001 ^†^
No	226 (76.4%)	345 (64.4%)	1	
Use of gloves at work				
Yes	204 (68.9%)	343 (64.0%)	1.3 (1–1.8)	0.083
No	89 (30.1%)	193 (36.0%)	1	

* cOR, Crude odds ratio; CI, confidence interval; ^†^ Significant difference, Chi-square *p*-value; ^‡^ Dispatch, cleaning, fire station, or plant.

## Data Availability

The datasets analyzed during this study are available from the corresponding author upon reasonable request.

## Appendix A

**Table A1:** Stratified analysis of individual factors associated with novel coronavirus disease in shift camps in Tengizchevroil, Kazakhstan during June–September 2020.

Characteristics		Stratum-Specific Odds Ratio (95% CI) * by Shift Camp					BD ^§^ *p*-value	LR ^¶^ *p*-value
	New Tengiz	Denkholm	Shanyrak	SK ^†^	Bolashak	Other ^‡^		
Male sex	1.9 (1.0–3.7)	0.4 (0.1–1.4)	1.9 (1.0–3.9)	2.2 (0.4–56.7)	0.4 (0.2–1.0)	0.6 (0.2–1.7)	0.217	0.223
Age group (≥ 36 years/ <36 years)	0.7 (0.3–1.3)	1.1 (0.6–2.0)	0.5 (0.3–0.9)	1.3 (0.5–3.6)	1.6 (0.7–3.7)	0.9 (0.4–2.1)	0.188	0.160
University degree vs. high school/college	2.1 (1.0–4.2)	2.0 (1.0–4.0)	2.2 (1.2–4.1)	0.9 (0.3–2.6)	0.6 (0.2–1.5)	0.5 (0.2–1.2)	0.014	0.082
Overweight (25.0–29.9 kg/m^2^)	1.2 (0.6–2.4)	1.2 (0.6–2.4)	1.0 (0.5–2.0)	1.3 (0.4–4.4)	0.8 (0.3–2.1)	0.8 (0.3–2.3)	0.972	0.958
Obesity (≥30 kg/m^2^)/ normal weight (<24.9)	1.3 (0.4–3.9)	3.4 (1.3–9.2)	0.8 (0.3–1.9)	1.3 (0.2–6.1)	2.5 (0.6–11.7)	0.7 (0.2–2.6)	0.197	0.112
Travel prior to coming to the shift	1.8 (0.6–5.2)	-	-	-	1.4 (<0.1,56.9)	0.7 (<0.1,9.2)	-	-
Social interaction outside of working hours	4.2 (1.9–9.3)	2.5 (1.3–4.9)	2.2 (1.2–4.2)	0.8 (0.3–2.3)	1.4 (0.6–3.3)	2.0 (0.8–4.7)	0.158	0.034
Fever ^#^	0.6 (0.3–1.4)	0.3 (0.1–0.5)	1.8 (0.8–4.9)	0.5 (0.2–1.5)	0.4 (0.2–1.0)	0.6 (0.2–1.7)	<0.001	0.028
Shortness of breath ^#^	0.8 (0.4–1.5)	0.6 (0.3–1.2)	3.2 (1.7–6.0)	0.4 (0.1–1.0)	0.2 (0.1–0.4)	1.3 (0.5–3.2)	<0.001	<0.001
Cough ^#^	1.0 (0.5–1.9)	0.4 (0.2–0.7)	2.1 (1.1–3.9)	0.5 (0.2–1.4)	0.2 (0.1–0.6)	1.0 (0.4–2.4)	<0.001	0.001
Loss of smell or taste ^#^	1.4 (0.8–2.7)	1.2 (0.6–2.3)	3.5 (1.8–6.8)	0.3 (0.1–0.9)	0.4 (0.2–0.8)	0.8 (0.3–1.9)	<0.001	0.001
Consider COVID-19 to be a serious issue	1.0 (0.4–2.2)	2.1 (1.1–4.2)	0.8 (0.4–1.8)	2.6 (0.7–8.5)	1.8 (0.7–5.2)	1.6 (0.5–5.9)	0.396	0.507
Believe that asymptomatic COVID-19 is contagious	0.5 (0.3–1.0)	0.3 (0.1–0.5)	1.2 (0.6–2.4)	0.6 (0.2–1.7)	0.2 (0.1–0.5)	0.7 (0.3–1.8)	0.007	0.005
Believe masks should be used outdoors	0.3 (0.1–0.6)	0.4 (0.2–0.8)	0.5 (0.2–0.9)	-	1.3 (0.4–4.0)	0.9 (0.4–2.5)	0.037	-
Believe masks should be used in public places	0.1 (<0.1–0.7)	0.5 (0.2–1.1)	1.8 (0.7–5.1)	-	0.4 (0.1–1.7)	0.1 (<0.1–0.6)	0.009	-
Believe masks should be used in a dormitory	0.1 (<0.1–0.4)	0.8 (0.4–1.7)	1.8 (0.7–5.1)	2.0 (0.3–50.9)	0.7 (0.2–2.7)	0.6 (0.1–2.1)	0.014	0.005
Used surgical masks	4.8 (2.5–9.5)	0.1 (0.1–1.0)	0.8 (0.3–2.0)	2.0 (0.3–50.9)	0.4 (0.1–1.2)	1.4 (0.3–12.2)	<0.001	0.001
Used respirators	15.3 (2.6–396.8)	1.0 (0.2–3.4)	0.9 (0.4–1.9)	0.8 (0.1–3.5)	0.3 (0.1–1.1)	0.9 (0.2–3.6)	0.007	0.094
Used fabric masks	0.1 (0.1–0.2)	0.7 (<0.1,6.2)	0.6 (0.1–3.3)	-	3.4 (1.2–10.1)	1.4 (0.2–8.5)	<0.001	-
3–4 masks vs. ≥ 5 changed per day	1.2 (0.5–2.8)	2.0 (1.0–4.1)	1.5 (0.7–3.2)	0.6 (0.2–2.5)	0.2 (0.1–0.7)	0.6 (0.2–1.6)	0.013	0.017
2 masks vs. ≥ 5 changed per day	9.1 (2.6–44.8)	1.9 (0.7–5.2)	0.7 (0.2–2.0)	0.9 (0.3–3.5)	8.3 (1.4–219.6)	1.2 (0.2–5.8)	0.013	0.001
1 mask vs. ≥ 5 changed per day	2.9 (0.5–17.3)	8.2 (0.9–243.8)	1.8 (0.5–6.6)	0.5 (<0.1–4.5)	1.2 (0.1–12.1)	–	0.349	-
Seldom use of hand sanitizers at the work vs. always	-	11.6 (5.1–28.5)	1.2 (0.3–5.3)	0.8 (0.2–3.5)	2.3 (0.3–21.7)	0.8 (0.1–8.5)	0.118	-
Non-use of hand sanitizers at the work vs. always	-	7.6 (2.7–22.8)	1.4 (0.4–6.0)	0.4 (0.1–1.9)	0.5 (0.1–5.0)	0.7 (0.1–6.8)	<0.001	-
Effect modification:	risk factor		protective factor		non-significant factor			
No effect modification:								

* CI, confidence interval; ^†^ SK = Senimdi kurylis; ^‡^ Other camps = Vengerka, Karat, and Birilik; ^§^ BD, Breslow-Day statistically significant test for odds ratio homogeneity within strata (BD *p*< 0.05); ^¶^ LR, Likelihood ratio *p*-value < 0.05 confirm that the effect of the variable is different across shift camps; ^#^ Knowledge of COVID-19 symptoms.

**Table A2:** Stratified analysis of environmental factors associated with novel coronavirus disease in Tengiz oil facility, assessing confounding and effect modification by shift camps during June–September 2020.

Characteristics		Stratum-Specific Odds Ratio (95% CI)* by Shift Camp					BD ^§^ *p*-value	LR ^¶^ *p*-value
	New Tengiz	Denkholm	Shanyrak	SK ^†^	Bolashak	Other ^‡^		
Pre-shift quarantine ≤ 7 vs. 8–14 days	4.0 (1.8–9.0)	1.4 (0.6–3.4)	0.3 (0.2–0.7)	0.1 (<0.1–0.6)	0.6 (0.2–1.7)	2.0 (0.7–6.8)	<0.001	0.001
Trained on COVID-19 prevention measures	0.1 (<0.1–0.3)	0.8 (0.3–1.9)	0.4 (0.1–1.0)	1.6 (0.4–12.1)	0.7 (0.1–3.2)	0.7 (0.2–2.3)	0.025	-
Contact with a COVID-19 patient at work (n = 756)	2.9 (1.4–5.9)	1.2 (0.5–2.6)	0.4 (0.2–0.7)	4.8 (0.5–34.7)	1.7 (0.7–4.3)	1.0 (0.4–2.5)	<0.001	0.019
Exposed to a COVID-19 patient with <1.5 m	0.4 (0.1–1.4)	9.4 (1.3–271.0)	5.4 (1.7–21.1)	-	1.7 (0.1–58.7)	0.5 (0.1–3.5)	0.009	-
Exposed to COVID-19 patient >15 min in the room	0.9 (0.2–4.0)	-	2.2 (0.5–16.6)	-	-	0.7 (0.1–4.9)	0.105	-
Sharing toilet on the floor of the dormitory	3.5 (0.9–14.7)	0.8 (0.4–1.4)	1.7 (0.9–3.4)	2.7 (0.9–10.5)	1.5 (0.2–8.9)	1.5 (0.6–3.6)	0.182	-
Individual toilet in the room	2.9 (0.7–12.6)	1.2 (0.6–2.2)	0.6 (0.3–1.1)	0.5 (0.1–1.3)	0.2 (<0.1–2.2)	0.6 (0.2–1.4)	0.304	-
Living with 1–4 neighbors vs. alone	0.2 (0.1–0.3)	0.3 (0.1–0.6)	0.4 (0.1–1.1)	1.1 (0.4–3.1)	1.0 (0.4–2.3)	0.5 (0.2–1.1)	0.008	0.033
Working in the infirmary/clinic	-	-	-	-	-	4.4 (0.9–34.9)	-	-
Transport work	1.1 (0.3–3.8)	-	1.2 (0.3–4.5)	2.6 (0.7–8.5)	1.4 (0.6–3.3)	0.7 (0.1–4.0)	0.076	-
Working in an office	4.8 (2.0–11.7)	1.4 (0.6–3.3)	1.3 (0.7–2.4)	0.5 (<0.1–2.7)	0.5 (0.2–1.3)	0.8 (0.3–1.9)	0.006	-
Working outdoors	3.8 (1.5–9.8)	0.3 (0.2–0.6)	1.2 (0.6–2.5)	0.9 (0.3–2.4)	0.5 (0.1–1.5)	0.8 (0.3–2.0)	<0.001	0.717
Working in a kitchen	0.3 (0.1–0.5)	5.6 (0.6–163.3)	0.2 (0.1–0.6)	1.6 (0.1–14.5)	2.5 (0.6–13.7)	2.1 (0.3–18.6)	<0.001	-
Working in a storeroom	-	0.5 (<0.1–4.0)	0.6 (<0.1–7.7)	-	-	-	0.344	-
Other work stations ^‡^	0.8 (0.4–1.6)	2.0 (0.9–4.8)	1.8 (0.8–4.2)	0.9 (0.2–3.1)	2.0 (0.4–11.2)	2.2 (0.6–9.6)	0.442	0.049
Maintaining 1.5 m distance at work	0.1 (<0.1–0.3)	0.4 (0.2–0.9)	0.4 (0.1–1.0)	0.2 (0.1–0.7)	2.1 (0.4–17.0)	1.3 (0.3–5.5)	0.008	-
Air conditioner at work	10.(1.6–286.0)	-	0.9 (0.2–3.8)	-	-	1.4 (<0.1–54.7)	0.050	-
Ventilation system at work	0.3 (0.1–0.8)	0.4 (0.1–1.8)	0.6 (0.3–1.2)	-	0.6 (0.1–2.4)	1.0 (0.2–5.3)	0.489	-
Availability of sanitizers at work	0.2 (<0.1–1.4)	10.0 (4.7–22.6)	3.0 (0.9–14.5)	1.0 (0.3–4.1)	1.0 (0.2–9.2)	3.5 (0.5–96.0)	<0.001	-
Use of gloves in the dormitory corridors	1.1 (0.5–2.5)	0.2 (0.1–0.5)	1.7 (0.7–4.1)	0.1 (<0.1–0.7)	0.4 (0.1–1.1)	2.0 (0.6–6.8)	<0.001	-
Used gloves on a bus	0.5 (0.2–1.4)	0.2 (0.1–0.4)	3.3 (1.5–7.9)	0.3 (0.1–0.9)	0.3 (0.1–0.8)	1.4 (0.4–4.7)	<0.001	0.018
Used gloves at work	0.2 (0.1–0.4)	13.1 (6.0–31.3)	0.8 (0.4–1.4)	0.4 (0.1–1.1)	2.0 (0.7–6.2)	2.4 (1.0–6.1)	<0.001	0.004
Effect modification:	risk factor		protective factor		non-significant factor			
No effect modification:								

* CI, confidence interval; ^†^ SK = Senimdi kurylis; ^‡^ Other camps = Vengerka, Karat, and Birilik; ^§^ BD, Breslow-Day statistically significant test for odds ratio homogeneity within strata (BD *p* < 0.05); ^¶^ LR, Likelihood ratio *p*-value < 0.05 confirm that the effect of the variable is different across shift camps.
